# Medical News

**Published:** 1853-04

**Authors:** 


### MEDICAL NEWS.

Dr. Drake's Work.—The preparation of the second volume of Dr. Drake's work "On the Diseases of the interior valley of North America," has been entrusted to Dr. S. Hanbury Smith of Cincinnati, a gentleman who, by his varied accomplishments and intimate relations with the deceased, is most admirably qualified for this undertaking. It will probably be completed next fall.

New York University.—We learn that Dr. M. Clymer has resigned the chair of Theory and Practice of Medicine in this Institution, and that Dr. John A. Swett has been appointed in his stead.

The Opium Trade.—We are pleased to receive a copy of the new edition of a valuable pamphlet on this subject by Dr. Nathan Allen, *of Lowell, Mass.*

We learn that he is preparing an article upon the abuse of opiates in Great Britian and the United States, and that he will be greatly obliged to merchants, druggists, or members of the medical profession who will communicate to his address, as given above, any facts bearing upon this subject.

The sixth annual meeting of the American Medical Association will be held in the city of New York on Tuesday, May 3, 1853.

The secretaries of all societies and other bodies entitled to representation in the association, are requested to forward to the undersigned correct lists of their respective delegations as soon as they may be appointed; and it is desired by the committee of arrangements that the appointments be made at as early a period as possible.

The following is an extract from Art. II of the constitution: "Each local society shall have the privilege of sending to the association one delegate for every ten of its regular resident members, and one for every additional fraction of more than half of this number. The faculty of every regularly constituted medical college or chartered school of medicine shall have the privilege of sending two delegates. The professional staff of every chartered or municipal hospital containing a hundred inmates or more, shall have the privilege of sending two delegates; and every other permanently organized medical institution of good standing shall have the privilege of sending one delegate."

EDW'D L. BEADLE,
*One of the Secretaries, No. 42 Bleecker st., New York.*

QUEEN'S COLLEGE FOR LADIES.—Medical students who diligently attend the courses of lectures prescribed by the licensing bodies, consider themselves well worked, although at no time are they advised to attend to more than four subjects during the same six months, or more than four lectures on the same day. And, in verity, to keep up the attention for four consecutive hours is no small mental exertion. We remember a teacher of medicine—himself in his early days one of the most distinguished students in one of the largest medical schools in the world—saying that six hours daily mental effort was as much as he ever knew a man capable of for any length of time, without injury to his mind or body. But, however different may be the opinions on this point in reference to men, we should have supposed, that, in regard to the weaker sex, the statement would have admitted of no question,—that all reasonable men would have been agreed on the impropriety of subjecting young females to a mental training exceeding in severity that imposed on students of medicine.

Our attention has been called to this subject by a physician who informs us, that he has had occasion to see more than one of the lady pupils at the Queen's College, in Harley street, in consequence of their having yielded to the temptations to over mental exertion held out to them at that institution.

The Queen's College is especially intended for young ladies qualifying themselves as governesses. The instruction is conveyed to the pupils by means of lectures, delivered by men of the highest reputation in the branches of science and literature they respectively teach.

Among the subjects discoursed on to these young ladies are Greek, Latin, Geometry, Algebra, Natural Philosophy, Astronomy, Mental and

Moral Philosophy, Logic, and Theology, Botany, Chemistry, Geology, and the useful arts ! ! !

For five or six hours in succession are girls, between the ages of 17 and 20, daily lectured to at the Queen's College on these subjects,—subjects, it will not be doubted, calling for as much reading, and as great mental exertion, as any comprised in the curricula of the Apothecaries' Society, the College of Surgeons, the College of Physicians, or the Universities of Dublin, Edinburgh, or London. Who can wonder that the result is what we are told it is ? A paper is now being extensively signed by the leading members of the Profession, expressive of their conviction, that a knowledge of the Principles of Physiology is most desirable for schoolmasters. May we not add, that a knowledge of cerebral physiology is essential for those who undertake the management of such institutions as that, on *one* fault of which we are now touching ?

It is really too bad, on the part of the Committee, to offer to those who lay the foundation of serious organic diseases in their "College," the beggarly reward of a certificate, stating that they are mentally qualified to undertake the duties of a governess. Why not enter into an arrangement with the Senate of the University of London, by which their pupils might obtain a Degree in return for their ruined health ? Perhaps a real advantage might follow—Female Bachelors and Masters of Arts, or rather Maids and Mistresses of Arts, might receive somewhat higher salaries, and enjoy a little more consideration in the families of the noble and wealthy than these certificated damsels ; they might possibly, then, be as highly estimated and as well paid as ladies' maids and cooks. With this, however, we have nothing to do ; but it is a duty we owe to society to call the attention of the Committee to the sacrifice of human health and life they are offering up, with no other practical result, that we can see, than the supplying those who require to have their children highly educated at a low price, with governesses who will do the work for which several masters ought to be paid.—*London Med. Times and Gaz.*

Dr. Marshall Hall.—Of course every medical reader is familiar with the name of this distinguished English author. He is now at Washington, accompanied by his lady and son. After visiting the South, he proposes to return and travel over the West extensively, and next season visit the Eastern States. Although considerably advanced in years, Dr. Hall is a laborious student, a close observer, and may justly be called one of the most celebrated medical writers of the age. We bespeak for him the attentions of the professional brotherhood wherever his steps may be directed.

Bulletin of Public Health.—Some alarming rumors have been in circulation at Paris: we may give our assurance, that all anxiety as to the state of the public health is without foundation. Information, upon the accuracy of which we may rely, authorises us to say that the choleriform affections which have presented themselves lately at the hospitals have not exceeded the number twelve during the last six months, and that such is about the usual proportion to other cases at this season. With one exception these cases were slight; the principal symptoms characteristic of Asiatic cholera were wanting, and the patients all recovered. One single circumstance was calculated to arouse public attention, namely, five cases of this nature presented themselves at the Hôtel Dieu upon the same day the week before last. The symptoms, however, yielded to proper treatment. The influenza (la grippe) has prevailed at Paris since the last fortnight of December, and has attacked, as usual, a large part of the population. As usual, too, but perhaps rather more strongly marked in the present epidemic than in any of the preceding, the disease has presented itself under two forms, a pectoral form, and an intestinal form. Under this last form it gives rise to colic, purging, to a prostration of strength most marked, to change in the features, to general loss of temperature, symptoms which recall, in reduced proportions, some of the phenomena of Asiatic cholera. But these accidents yield wonderfully to the employment of some diffusible stimulus, viz., infusions of peppermint and chamomile, and to the administration of opium in draughts or lavements. Opium is most useful in arresting these symptoms, which do not resist treatment nearly so much as those accompanying the pectoral form of "la grippe," the progress of which, usually without danger, it is difficult to stop.

It is very true there are, at the present moment, a great number of sick in the hospitals; supplemental beds have been put into the Hôtel Dieu, and it is feared a yet larger number will be required. This fact arises from two causes. First, the suppression of the temporary hospital of Bon Secours; secondly, the exceeding mildness of the winter, which has permitted the continuance of the immense works now in progress at Paris, and has consequently retained a considerable number of laborers who usually go back at this season to their homes. To resume, in spite of "la grippe," in spite of the crowded state of the hospitals, where there are many cases of typhoid fever, nothing justifies the apprehensions spread throughout the public mind. Asiatic cholera does not exist in Paris. It is retiring daily from central Europe, and every thing confirms us in the hope, which we expressed last November, that the epidemic of Poland and Russia would die in the place of its birth.—*London Med. Times. and Gaz.*, from *Union Médicale.*

History of Corsets. By M Bouvier.—The Academy of Medicine (Séance du 25 Janvier, 1853, Presidence de M. Bérard) feeling with propriety that no subject affecting the health is below consideration, has given its attention to a report from M. Bouvier upon ladies' stays. The work is divided into two parts; the first, now before the public, being the history of stays. The report bears especially upon stays without seams and without a mechanical busk. The learned author, who seems to have ransacked both ancient and modern history for information upon so absorbing a matter, arrives at the following conclusions.

1. The history of the dress of the principal people of antiquity shows that the want of a retentive garment, more or less constricting, round the trunk in the female was felt in ancient as well as in modern Europe.

2. In other times, as now, women have been disposed to overdo this circular constriction, to the detriment of their health.

3. In the history of modern civilization, one sees, after the relinquishment of the ample tunic of the Roman ladies, the figure first simply surrounded in a well-fitting corsage; then inclosed and bound in a sort of cuirass, called "corps à'baleines;" and, lastly, brought out and supported by the present corset, the last form of this special garment.

4. Although corsets, when improperly employed, may be prejudicial, yet, when well made and well adjusted, they have not the injurious effects usually ascribed to them.

5. It is an error to attribute the constriction of the lower part of the chest to the influence of stays. A constriction is normal, within certain limits, in both sexes, and subject to vary from other causes than the pressure exercised by this article of dress.

6. There is no proof that the use of corsets produces deformity of the vertebral column.

7. Not only should motives deduced from asthetics and from the social destination of woman induce the physician to permit the use of corsets, under proper restrictions, but, moreover, there are many circumstances, such as the volume of the bosom, the relaxation or the distension of the muscular wall of the abdomen, the habitual bending of the trunk, the lateral deviation of the spine, etc., which give formal indications for the employment of this sort of bandage, whether upon hygienic principles, or as an aid to cure certain lesions.

The second part of this contribution to medical literature is to be presented at the next *séance.—London Med. Times. and Gaz.*, from *Union Médicale.*

